# Health education actions on male breast cancer: A protocol for systematic review and meta analysis

**DOI:** 10.1097/MD.0000000000030931

**Published:** 2022-10-21

**Authors:** José Felipe Costa da Silva, Gilson de Vasconcelos Torres, Luciana Araújo dos Reis, Julliane Tamara Araújo de Melo Campos, Vilani Medeiros De Araújo Nunes, Jéssyca Camila Carvalho Santos, Thalia Natasha Silva Barbalho, Thaiza Teixeira Xavier Nobre

**Affiliations:** a Graduate Program in Public Health, Federal University of Rio Grande do Norte, Natal/RN, Brazil; b Nursing Department, Federal University of Rio Grande do Norte, Natal, RN, Brazil; c Jequié College of Nursing, State University of Southwest Bahia, Jequié, BA, Brazil; d Morphology Department, Biosciences Center, Federal University of Rio Grande do Norte, Natal, RN, Brazil; e Department of Public Health, Federal University of Rio Grande do Norte, Natal, RN, Brazil; f Graduating in Physiotherapy, College of Health Sciences of Trairi, Federal University of Rio Grande do Norte, Santa Cruz-RN, Brazil; g Master in Public Health, College of Health Sciences of Trairi, Federal University of Rio Grande do Norte, Santa Cruz-RN, Brazil; h College of Health Sciences of Trairi, Federal University of Rio Grande do Norte, Santa Cruz-RN, Brazil.

**Keywords:** health education, health promotion, male breast cancer, protocol, systematic review

## Abstract

Although rare, the rates of diagnosis of male breast cancer (MBC) have been increasing over the years, and it can be quite aggressive in the male organism. Some strategies such as health education disseminating knowledge about MBC can be essential for early discovery and finding better prognosis.

**Objective:**

Protocol to map the available evidence of health education approaches on MBC.

**Methods:**

A scoping review on health education on MBC will be carried out in Latin American and Caribbean Literature on Health Sciences (LILACS), Web of Science, Scopus, Scielo, Online System of Literature Search and Analysis Medical (MEDLINE), Embase, Virtual Health Library (VHL). Two independent reviewers will perform screening, data extraction, and risk of bias assessment through the Joanna Briggs Institute (JBI) Critical Assessment Checklist. For the quality of evidence, the Grading of Recommendations, Assessment, Development and Evaluation (GRADE) Analysis will be used.

**Results:**

The results of this review will be published in a peer-reviewed journal.

**Conclusions:**

This scoping review will provide evidence of how health education on MBC is being addressed in health systems. Evidence can help healthcare professionals and patients recognize the most effective educational inventions in disseminating knowledge and preventing MBC.

## 1. Introduction

Male breast cancer (MBC) is a rare disease corresponding to about 1% of all breast carcinomas diagnosed in the United States annually. The risk of developing this type of cancer increases with advancing age, it is currently known that, throughout their lives, about 1:1000 men can be diagnosed with this problem, while the prevalence in women is more around 1:8.^[1,2]^

Over the years, the incidence of MBC has been on the rise on all continents, worldwide, newly diagnosed cases have increased from 8500 in 1990 to 23,100 in 2017, and these figures may be even higher due to the invisibility of the topic. Some risk factors related to these numbers include increased longevity, obesity, diseases, testicular tumors, and gene mutations such as BRCA2.^[3,4]^

The presentation of MBC is usually the presence of a painless volume in the breast and armpit region, wrinkled skin, nipple retraction and increase in the size of the axillary lymph nodes. Despite the small risk for males to develop the disease, when diagnosed, this cancer is more aggressive and is in an advanced stage, contributing to a worse prognosis and lower survival when compared to cases in women. The treatment is similar to that for women and may involve surgery, radiotherapy and/or chemotherapy.^[5,6]^

Health promotion and specific prevention strategies such as mammography are effective in the early screening of female breast cancer. In the case of men, there is no recommendation for screening at detection due to the low general prevalence of the disease, this recommendation is extended even to men with known risk. In men’s health care, this topic is not commonly addressed, being absent in health actions and services.^[7,8]^

Knowledge of MBC is essential for early diagnosis as well as for structuring of prevention and health promotion strategies. Despite this urgent need, since cases are increasing every year, research carried out with male college students shows that only one-third knew about the existence of MBC and even smaller proportions were aware of risk factors and the main signs and symptoms. Evidence from this study suggests that undergraduate students have limited knowledge about breast cancer in men.^[9]^

Health education actions for health professionals and the target population are necessary for better prevention of MBC, since there are prejudices, stigmas and lack of prior knowledge that hinder early diagnosis actions. These must happen mainly at the primary level, for the main means of prevention to be included in the health systems. In this context, this review is justified by the need to understand how interventions are carried out at the level of health education on MBC.^[10]^

A preliminary search was conducted at PROSPERO and no current or ongoing scoping reviews or systematic reviews on the topic were found. In this context, the objective of this protocol was to map the available evidence on health education actions on MBC.

### 1.1. Research question

What are the main methods, instruments or strategies used in health education actions on male breast cancer?

### 1.2. Inclusion criteria

#### 1.2.1. Participants

Studies with participants over 18 years of age without age or sex restrictions, who may be users of the health system or health professionals, will be included.

#### 1.2.2. Concept

This review will consider studies that explore health education actions and describe their educational strategies for approaching the topic of MBC. It will also describe the positive impacts on prevention and early diagnosis of MBC.

#### 1.2.3. Context

The present review will mainly consider studies focusing on the male and elderly population and health education interventions, not limited to these aspects, as well as any geographic region.

#### 1.2.4. Types of studies

This scoping review will consider studies with quantitative, qualitative, and mixed methods designs for inclusion. In addition, systematic reviews and text and opinion articles will be considered for inclusion in the scope review proposal.

## 2. Methods

This is a research protocol for a scoping review following the recommendations of the Reviews extension for Scoping Reviews (PRISMA-ScR)^[11]^ proposed by the Joanna Briggs Institute.^[12]^ The protocol of this study was registered in the Open Science Framework (https://osf.io/5qs9k/).

### 2.1. Search strategies

The search strategy will aim to locate published and unpublished primary studies, reviews and text, and opinion articles. A limited initial search in the Web of Science was performed to identify articles on the topic. The text words contained in the titles and abstracts of relevant articles and the indexing terms used to describe the articles were used to develop a comprehensive search strategy as can be seen in the supplemental digital content (Appendix I, Supplemental Digital Content, http://links.lww.com/MD/H455), where the search strategies in the Web Of Science research database conducted in March 2022 are presented. The search strategy, including all the identified keywords and indexing terms, will be adapted for each information source included. Reference lists of articles selected for full-text review included in the review will be selected as additional articles.

There will be no language or time limitations.

Databases to be searched include

MEDLINE (Ovid Online - www.ovid.com);

EMBASE (Ovid Online - www.ovid.com);

PubMed (www.ncbi.nlm.nih.gov/pubmed/);

LILACS (lilacs.bvsalud.org/);

SCIELO (www.scielo.org/php/index.php);

CINAHL (www.ebscohost.com);

SCOPUS (via capes periodical portal);

WEB OF SCIENCE (via capes periodical portal);

EMBASE (Ovid Online - www.ovid.com);

Virtual Health Library (via capes periodical portal).

Sources of unpublished studies and gray literature to be searched include Catalog of Theses and Dissertations from Brazil;

Open Access Scientific Repository of Portugal;

Gray Literature Information System in Europe (Open Grey).

### 2.2. Selection of studies

After the search, all identified records will be grouped and loaded into all data and will be exported from databases in EndNote, Export, Refman/RIS or Text format and included in the Rayyan QCRI platform.^[13]^ This online platform was developed by the Qatar Computing Research Institute, available for free, offers a wide range of resources, including uploading citations in different formats, navigations, automatic extractions to exclude duplicate citations. For this study, the Rayyan QCRI will be used for exclusion of duplicated articles and it will be required for title and abstract analysis. After a pilot test, titles and abstracts will be selected by 2 independent reviewers for evaluation according to the inclusion criteria for the review.

The full text of selected citations will be evaluated in detail according to the inclusion criteria by 2 independent reviewers. The reasons for excluding full-text articles that do not meet the inclusion criteria will be recorded and reported in the scoping review. Any disagreements that may eventually arise between reviewers at each stage of the selection process will be resolved by discussion or with a third reviewer. The research results will be reported in full in the final scoping review and presented in a PRISMA flow diagram^[14]^ as can be seen in the supplemental digital content (Appendix II, Supplemental Digital Content, http://links.lww.com/MD/H456) that presents the PRISMA 2020 flow diagram for new systematic reviews which included searches of databases and registers only.

### 2.3. Data extraction

Data will be extracted from articles included in the scoping review by two independent reviewers using a data extraction tool developed by the reviewers in Excel spreadsheet format. The extracted data will include specific details about the identification of the publication containing the author/year information; country of study, language; journal; study design; participants; type of intervention; educational strategies; intervention and conclusion site. This extraction will be performed by the two reviewers and then compared, if no agreement is reached, the third reviewer will be called. The draft data extraction tool will be modified and revised as necessary during the data extraction process for each article included. Modifications will be detailed in the full scoping review.

A draft extraction tool is provided in the supplemental digital content (Appendix III, Supplemental Digital Content, http://links.lww.com/MD/H457) this extraction box contains the key information to be extracted from the included studies.

### 2.4. Data analysis and presentation

Data will be descriptively mapped and presented through tables and figures with the distribution of articles by publication period, country, and main variables found. A narrative summary of the overall results will accompany tables and figures that describe key health education strategies in MBC.

### 2.5. Ethics and dissemination

For the development of this study, approval of ethics and consent is not necessary because it is a systematic review that will use secondary studies.

## 3. Discussion

The adequate inclusion of health education actions should be priority for an early diagnosis of various types of cancer. In the case of MBC, this should be addressed within the context of men’s health. As it is a subject usually discussed among the female audience, more assertive guidance is necessary in order to reach men with risk factors as well as health professionals.^[15]^

A low adherence and search for health care is known to happen among men, which may be explained by reasons of gender, time incompatibility, fear of detecting serious illnesses, insufficient number of consultations in public health services and professionals, and lack of specialists. These data may be even lower when the male body is affected with a pathological process common in females, leading to a process of rejection of the sick body. Men have difficulty accepting MBC, leading to serious mental illness.^[16,17]^

Personal and environmental aspects must be considered when health care is linked to cancer. Psychological aspects are usually affected, placing the individual in a vulnerable situation, further increasing the chances of illness and death. Greater knowledge, management, and preparation of the professionals who assist these patients and their relatives are necessary, especially where male patients with breast cancer have worse survival rates compared to female patients.^[18,19]^

It is expected that by concluding this review, the main findings may contribute to improvements in health education processes for professionals and users of health services, aiming at enhancing the knowledge and dissemination of the existence of MBC and providing subsidies on how to approach this topic in the daily practice.

## Author contributions

**Conceptualization:** José Felipe Costa da Silva.

**Methodology:** Vilani Medeiros De Araújo Nunes, Gilson de Vasconcelos Torres.

**Project administration:** Gilson de Vasconcelos Torres.

**Supervision:** Luciana Araújo dos Reis, Julliane Tamara Araújo de Melo Campos.

**Writing – original draft:** José Felipe Costa da Silva, Thaiza Teixeira Xavier Nobre, Jéssyca Camila Carvalho Santos, Thalia Natasha Silva Barbalho.

**Writing – review & editing:** Julliane Tamara Araújo de Melo Campos.

## Supplementary Material


