# Supplementary material for: Health education actions on male breast cancer: A protocol for systematic review and meta analysis
Source: Medicine (Baltimore). 2022 Oct 21;101(42):e30931. doi: 10.1097/MD.0000000000030931 (PMC9592352; doi:10.1097/MD.0000000000030931)
Supplement: Supplementary file 1 [file medi-101-e30931-s001.pdf]

Appendix I: Search strategies

WEB OF SCIENCE (via capes peridocal portal), research conducted in March 2022.

**Search**	**Query**	**Records retrieved**		
#1	“Breast Cancer, Male” OR “Breast Carcinoma, Male” OR “Breast Neoplasm, Male” OR “Breast Tumor, Male” OR “Breast Tumors, Male” OR “Cancer, Male Breast” OR “Carcinoma, Male Breast” OR “Male Breast Cancer” OR “Male Breast Carcinoma” OR “Male Breast Neoplasm” OR “Male Breast Neoplasms” OR “Male Breast Tumor” OR “Male Breast Tumors” OR “Neoplasm, Male Breast” OR “Neoplasms, Breast, Male” OR “Neoplasms, Male Breast” OR “Tumor, Male Breast” OR “Tumors, Breast, Male” OR “Tumors, Male Breast”	2,183		
#2	“Health Education” OR “Community Health Education” OR “Education, Community Health” OR “Education, Health” OR “Health Education, Community”	45,785		
#3	#1 AND #2	5.933		
There were no limitations as to language, publication date or geographic area.				
