# Supplementary material for: Health education actions on male breast cancer: A protocol for systematic review and meta analysis
Source: Medicine (Baltimore). 2022 Oct 21;101(42):e30931. doi: 10.1097/MD.0000000000030931 (PMC9592352; doi:10.1097/MD.0000000000030931)
Supplement: Supplementary file 2 [file medi-101-e30931-s002.pdf]

Appendix II: PRISMA 2020 flow diagram for new systematic reviews which included searches of databases and registers only

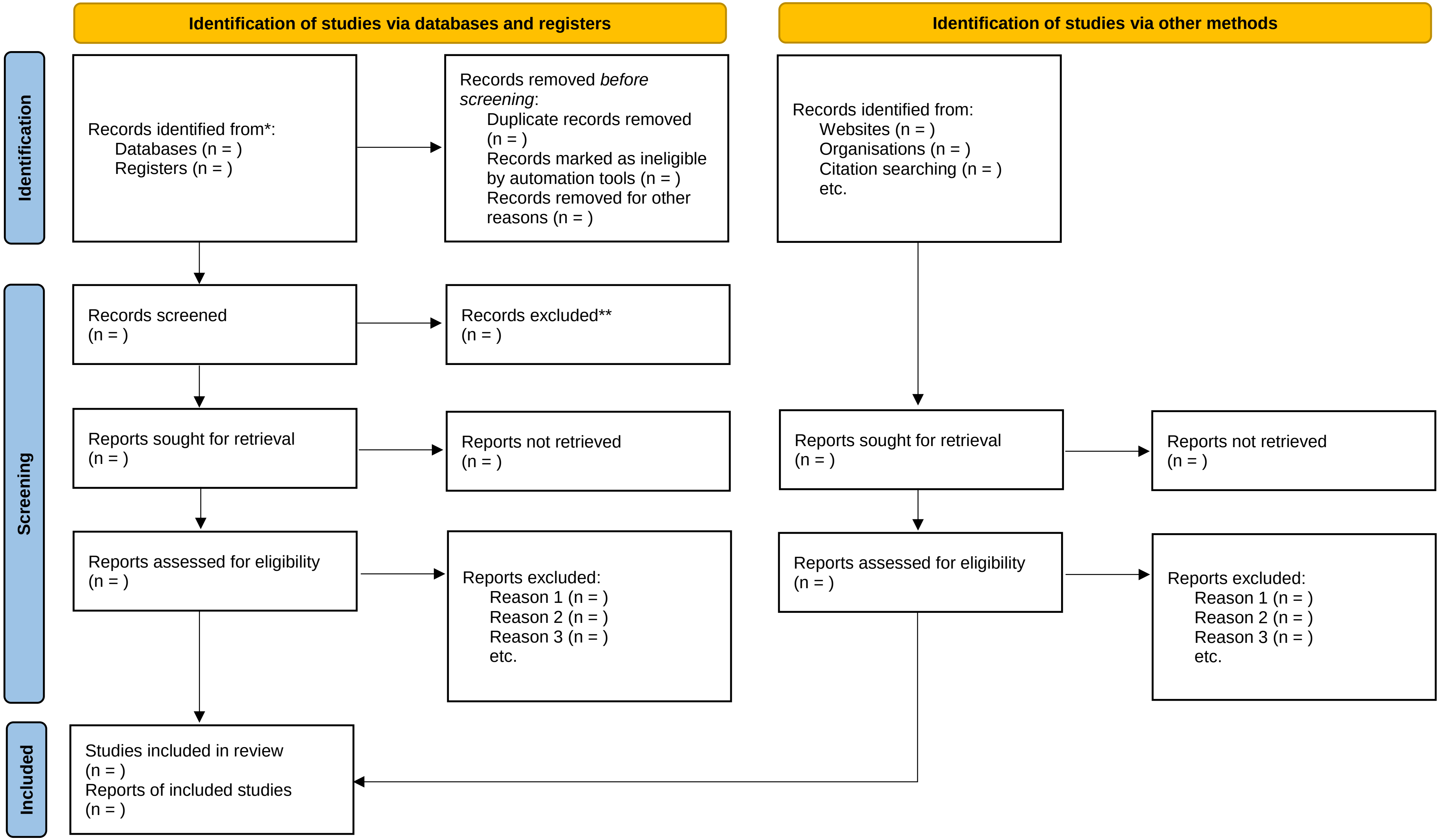

*Consider, if feasible to do so, reporting the number of records identified from each database or register searched (rather than the total number across all databases/registers).
**If automation tools were used, indicate how many records were excluded by a human and how many were excluded by automation tools.

From: Page MJ, McKenzie JE, Bossuyt PM, Boutron I, Hoffmann TC, Mulrow CD, et al. The PRISMA 2020 statement: an updated guideline for reporting systematic reviews. BMJ 2021;372:n71. doi: 10.1136/bmj.n71. For more information, visit: http://www.prisma-statement.org/
