# Supplementary material for: Health education actions on male breast cancer: A protocol for systematic review and meta analysis
Source: Medicine (Baltimore). 2022 Oct 21;101(42):e30931. doi: 10.1097/MD.0000000000030931 (PMC9592352; doi:10.1097/MD.0000000000030931)
Supplement: Supplementary file 3 [file medi-101-e30931-s003.pdf]

Appendix III: Data extraction tool

Author/ Year	Country	Language	Journal	Study design	Participants	Intervention	Strategies	Place of intervention	Conclusion
